# How obsessive–compulsive and bipolar disorders meet each other? An integrative gene-based enrichment approach

**DOI:** 10.1186/s12991-020-00280-9

**Published:** 2020-05-09

**Authors:** Sajedeh Hamidian, Abbas Pourshahbaz, Ali Bozorgmehr, Esmaeil Shahsavand Ananloo, Behrooz Dolatshahi, Mina Ohadi

**Affiliations:** 1 Department of Clinical Psychology, University of Social Welfare and Rehabilitation Sciences (USWR), Tehran, Iran; 2 Iran Psychiatric Hospital, Iran University of Medical Sciences (IUMS), Tehran, Iran; 3 Department of Psychosomatic, Imam Khomeini Hospital Complex, School of Medicine, Tehran University of Medical Sciences (TUMS), Tehran, Iran; 4 Iranian Research Center on Aging, University of Social Welfare and Rehabilitation Sciences (USWR), Tehran, Iran

**Keywords:** Enrichment analysis, Psychiatric genetic, Obsessive–compulsive disorder, Bipolar disorder, Genetic network

## Abstract

**Background:**

The novel approaches to psychiatric classification assume that disorders, contrary to what was previously thought, are not completely separate phenomena. In this regard, in addition to symptom-based criteria, disturbances are also considered on the basis of lower level components. With this viewpoint, identifying common biochemical markers would be beneficial in adopting a comprehensive strategy for prevention, diagnosis and treatment.

**Main body:**

One of the problematic areas in clinical settings is the coexistence of both obsessive–compulsive disorder (OCD) and bipolar disorder (BD) that is challenging and difficult to manage. In this study, using a system biologic approach we aimed to assess the interconnectedness of OCD and BD at different levels. Gene Set Enrichment Analysis (GSEA) method was used to identify the shared biological network between the two disorders. The results of the analysis revealed 34 common genes between the two disorders, the most important of which were *CACNA1C*, *GRIA1*, *DRD2*, *NOS1*, *SLC18A1*, *HTR2A* and *DRD1*. Dopaminergic synapse and cAMP signaling pathway as the pathways, dopamine binding and dopamine neurotransmitter receptor activity as the molecular functions, dendrite and axon part as the cellular component and cortex and striatum as the brain regions were the most significant commonalities.

**Short conclusion:**

The results of this study highlight the role of multiple systems, especially the dopaminergic system in linking OCD and BD. The results can be used to estimate the disease course, prognosis, and treatment choice, particularly in the cases of comorbidity. Such perspectives, going beyond symptomatic level, help to identify common endophenotypes between the disorders and provide diagnostic and therapeutic approaches based on biological in addition to the symptomatic level.

## Introduction

Obsessive–compulsive disorder (OCD), a devastating neuropsychiatric disorder with a strong genetic basis, affects 1–3% of the general population [1]. OCD is mainly characterized by repetitive, intrusive thoughts, images and impulses called obsessions and repetitive ritualistic behaviors, called compulsions [2, 3]. A great number of patients with OCD experience cyclical courses manifested by episodes of recurrence and remission [4]. This sinusoid phenomenon somewhat resembles the course of bipolar disorder (BD), another chronic psychiatric situation with cyclical episodes of mania and depression. Studies have provided evidence that there is a high rate of comorbidity between the two disorders. For instance, BD is reported to be diagnosed in 12–23% of clinical patients with OCD [5, 6]. Remarkably, D’Ambrosio et al. have found that cyclothymia is the dominant temperament in 54% of patients with OCD [7]. On the other hand, 21–35% of patients diagnosed with BD are reported to manifest OCD core symptoms [8].

Although in terms of Feinstein thesis, comorbidity is defined as the co-existence of diagnostic criteria of two or more disorders in one subject [9], the relationship between OCD and BD is beyond this simple definition. The co-occurring symptoms of the two disorders initially attracted clinicians’ attention [10, 11]. It was then observed that OCD and BD comorbidity is often accompanied by other conditions such as post-traumatic stress disorder (PTSD), eating disorder agoraphobia, panic disorder, earlier age of onset and a greater number of major depressive episodes [7, 10]. Besides, major psychiatric disorders are very debilitating, as Ghio et al. have showed in major depressive disorder (MDD), early diagnosis and treatment can significantly reduce the unfavorable outcomes including disability of the disorder [12]. Thus identifying the paths towards them are essential for early and timely treatment [13].

Moreover, the evidence suggests a high rate of co-inheritance of the two disorders. For example, families of patients with OCD have a higher prevalence rate of BD than the controls [14] and BD patients with family history of mood disorders had a significantly higher lifetime prevalence of OCD [15]. Additionally, having OCD is reported to increase the risk of BD 13-fold greater than not having OCD [16].

Although the two disorders do not share much at the phenotypic and behavioral levels, common endophenotypes have tightened the link between them. For example, both patients with BD and OCD showed impaired performance in verbal episodic memory mediated by semantic clustering abilities [17]. Deficient response inhibition and sustained attention are the other cognitive endophenotypes observed in both patients with OCD [18–20] and BD [21]. Furthermore, Benatti et al. reported significantly higher impulsivity levels in OCD patients compared to the controls [22], whereas, impulsivity is a well-known criterion for predicting manic episodes and onset of BD [23]. This moderately heritable component remains elevated during euthymia in BD and has been found among relatives of BD patients as well [15, 24, 25]. Adida et al. reported a trait-related impairment in decision-making in patients with BD compared to the healthy controls [26], while the addiction model emphasizes on risky decision-making behaviors in the psychopathology of OCD [27].

At the anatomical level, the brain regions involved in BD and OCD are highly overlapped. While hypo-activity in the orbitofrontal cortex (OFC) and dorsolateral prefrontal cortex (DLPFC) is suggested to be associated with manic episodes [28], parallel studies have demonstrated hyperactivity in the mentioned regions in patients with OCD as well [29]. Furthermore, anterior cingulate cortex (ACC) and striatum are the other shared brain regions involved in the pathophysiology of both disorders [30, 31].

More recently, genome-wide association studies (GWASs) and a significant number of confirmative candidate gene association studies, have identified shared loci for BD and OCD [32–35]. In a recent study, O’Connell et al. reported common genetic etiology for schizophrenia (SCZ), BD, autism spectrum disorders (ASD) and OCD. They suggested more researches on cross-disorder shared components in order to obtain translatable results for more specified clinical applications, prognosis and treatment management in mental health settings [36].

Although BD and OCD are phenotypically recognized as two distinct disorders, studies have reported high comorbidity of them. Existing theories have had little success in explaining the causes of this comorbidity phenotypically and sometimes they contradict each other; hence, we hypothesized that this comorbidity is rooted in the genetic similarities between the two disorders. Based on our knowledge, it seems that there is no comprehensive and integrative study on the shared genetic basis of OCD and BD yet. Therefore, the aim of the present study was to employ an exploratory approach to characterize the shared genetic basis of BD and OCD using a gene set-based approach following by molecular, cellular and pathway enrichment analyses. We assume that the OCD may share common genetic etiological factors and biological processes with BD.

## Methods

### Gene finding

In order to obtain genes significantly associated with OCD and BD, we searched the PubMed and Google Scholar databases manually, using related keywords. The keywords were combination of: “bipolar disorder”, “BD”, “manic-depressive disorder”, “bipolar”, “mania”, “obsessive–compulsive disorder”, “OCD”, “gene”, “association”, “linkage”, “meta-analysis”, “genome-wide association study”, “genome-wide”, “GWAS”, “exome wide” and “polymorphism”. The genes retrieved from human structural genetic studies, and the genetic database for OCD [37].

Studies that had found genes associated with OCD symptoms, manic phases without BD diagnosis, some common features in the two disorders such as suicidal behavior, as well as gene expression studies were excluded. Association structural genetic studies were included regardless of age, ethnicity and gender of the evaluated individuals and also regardless of the published year. Linkage studies were included provided that the name of the gene was mentioned in the intended loci and the found marker was at the maximum distance equal to 500 base pair from the mentioned gene. In order to prevent the repetition of genes with different names, the Ensembl ID of each gene was obtained through Ensembl genome browser 92. Finally, a list of 397 BD genes and 148 OCD genes were achieved. Fifty-eight genes were shared between the two disorders and were considered for further analysis. The final list of included genes is presented in Additional file 1.

### Gene Set Enrichment Analysis

Gene Set Enrichment Analysis (GSEA) is a powerful method for interpreting the biological meaning of a list of genes or proteins that provide important insights into the biological mechanisms underlying that gene set. This method uses the proportion-based statistical approaches to identify certain molecular functions, cellular components or biological processes which are over- or under-represented within the lists of interest.

We used WebGestalt (WEB-based Gene SeT AnaLysis Toolkit) for the gene set enrichment analysis. WebGestalt supports three well-established methods for enrichment analysis, including Over-Representation Analysis (ORA), Gene Set Enrichment Analysis (GSEA), and Network Topology-based Analysis (NTA). It incorporates information from different public resources and provides an easy way to make sense out of gene lists. In our analysis, we used ORA method for pathway enrichment analysis (based on the KEGG database [38]), and molecular functions and cellular component enrichment analysis (based on the Gene Ontology [GO]) Consortium database: (http://www.geneontology.org/) [39]. Moreover, Cell Type-Specific Expression Analysis (CSEA) tool was used for identifying candidate circuits and regions, by using a list of genes that have the most expression in their cells (http://genetics.wustl.edu/jdlab/csea-tool-2/).

The resulting data provided by enrichment analysis was analyzed and visualized using Cytoscape v3.6.0. Cytoscape is an open source software platform for complex molecular interaction network visualization and data integration. Using Network Analyzer toolbox for Cytoscape, a broad set of network topological parameters (number of nodes, edges and connected components, betweenness centrality and closeness centrality) were created. In the present diagram, the sizes of the nodes are based on betweenness centrality. Betweenness centrality is a measure of centrality in a graph based on shortest paths that measures the extent to which a vertex lies on paths between other vertices.

## Results

**Fig. 1:**
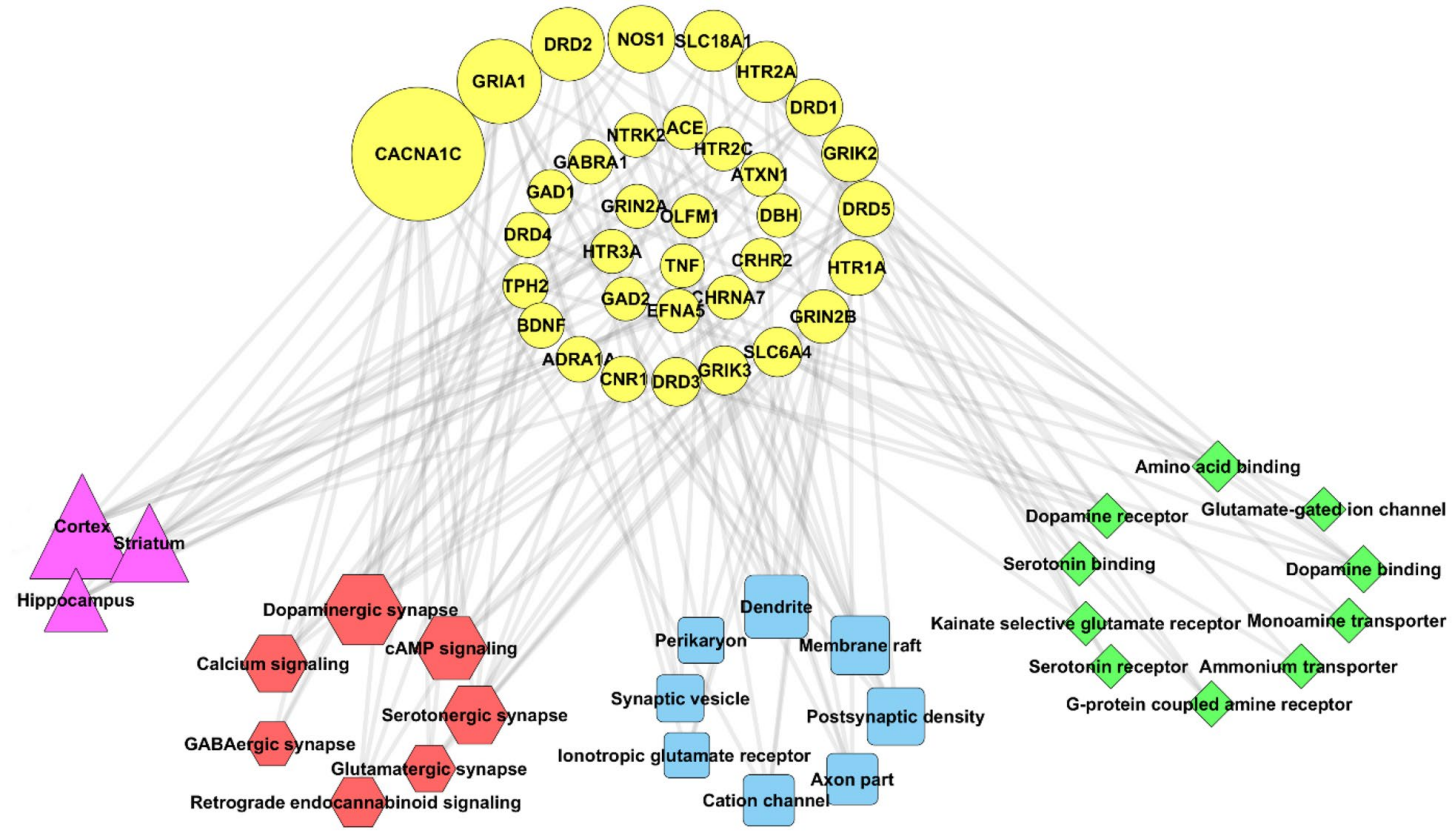
The analyzed network of features involved in the pathogenesis of both OCD and BD. The yellow circles represent the genes, green diamonds the molecular functions, blue squares the cellular components, red hexagons the pathways and violet triangles represent brain regions. The larger the nodes, the bigger the betweenness centrality

The reconstructed interactive network consisted of 62 nodes and 128 edges, including 34 genes, 10 molecular functions, 7 pathways, 8 cellular components and 3 brain regions. On average, each gene was involved in 4 molecular functions, 5 pathways, 5 cellular components, and 3 brain regions (Fig. 1).

**Fig. 2:**
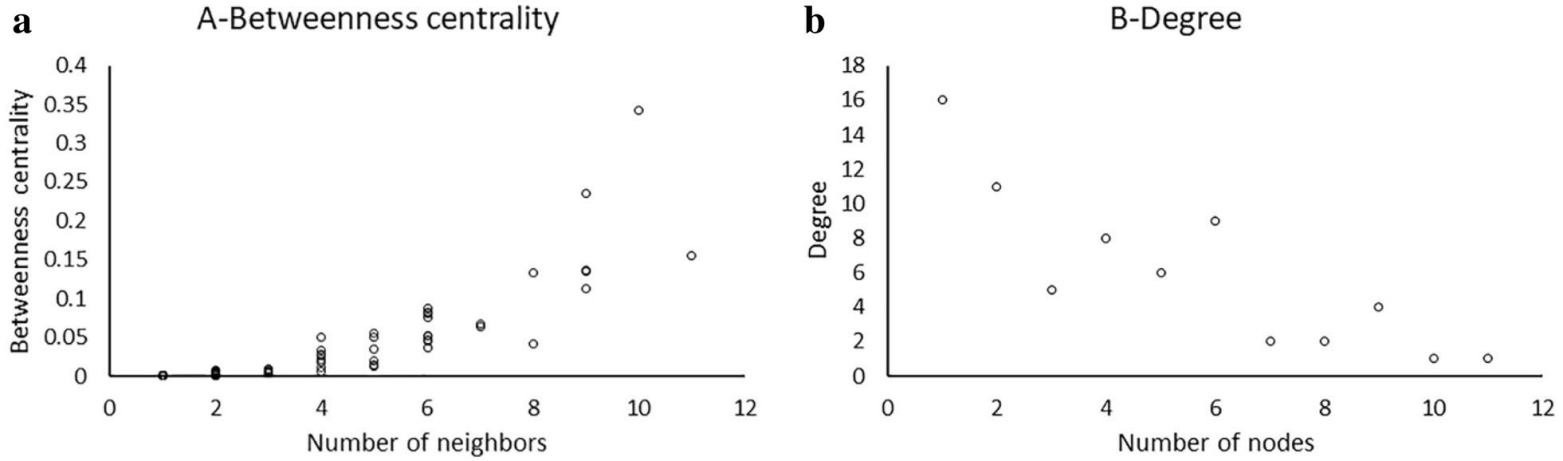
Topological characteristics of the reconstructed network. **a** Betweenness centrality distribution of the nodes in the network. **b** Node degree distribution in the network

Topological analysis of the network demonstrated that the majority of the nodes have the centrality between 0 and 0.1 (Fig. 2a). Furthermore, it was found that the majority of the nodes have a degree of connection between 1 and 2 (Fig. 2b).

*CACNA1C*, *GRIA1*, *DRD2*, *NOS1*, *SLC18A1*, *HTR2A* and *GRIK2* were the most central genes in the network, respectively. The other central genes in the network are shown in Table 1.

**Table 1:** The results of enrichment analysis for genes involved in the pathogenesis of both OCD and BD

	Gene full official name	HGNC symbol	Betweenness centrality
1	Calcium voltage-gated channel subunit alpha1 C	CACNA1C	0.338153
2	Glutamate ionotropic receptor AMPA-type subunit 1	GRIA1	0.1574122
3	Dopamine receptor D2	DRD2	0.1136524
4	Nitric oxide synthase 1	NOS1	0.0903783
5	Solute carrier family 18 member A1	SLC18A1	0.0661232
6	5-Hydroxytryptamine receptor 2A	HTR2A	0.0660831
7	Dopamine receptor D1	DRD1	0.0500920
8	Glutamate ionotropic receptor kainate-type subunit 2	GRIK2	0.0481045
9	Dopamine receptor D5	DRD5	0.0458659
10	5-Hydroxytryptamine receptor 1A, serotonin receptor	HTR1A	0.0443705
11	Glutamate ionotropic receptor NMDA-type subunit 2B	GRIN2B	0.036738
12	Solute carrier family 6 member 4	SLC6A4	0.020317
13	Glutamate ionotropic receptor kainate-type subunit 3	GRIK3	0.038491
14	Dopamine receptor D3	DRD3	0.017168
15	Cannabinoid receptor 1	CNR1	0.007217
16	Adrenoceptor alpha 1A	ADRA1A	0.006255
17	Brain-derived neurotrophic factor	BDNF	0.005617
18	Tryptophan hydroxylase 2	TPH2	0.0045508
19	Dopamine receptor D4	DRD4	0.004232
20	Glutamate decarboxylase 1	GAD1	0.003155
21	Gamma-aminobutyric acid type A receptor alpha1 subunit	GABRA1	0.001786
22	Neurotrophic receptor tyrosine kinase 2	NTRK2	0.001023
23	Angiotensin I converting enzyme	ACE	0
24	Ataxin 1	ATXN1	0
25	Cholinergic receptor nicotinic alpha 7 subunit	CHRNA7	0
26	Corticotropin-releasing hormone receptor 2	CRHR2	0
27	Dopamine beta-hydroxylase	DBH	0
28	Ephrin A5	EFNA5	0
29	Glutamic acid decarboxylase 2	GAD2	0
30	Glutamate ionotropic receptor NMDA-type subunit 2A	GRIN2A	0
31	5-Hydroxytryptamine receptor 2C	HTR2C	0
32	5-Hydroxytryptamine receptor 3A	HTR3A	0
33	Olfactomedin 1	OLFM1	0
34	Tumor necrosis factor	TNF	0

Among the pathways found through enrichment analysis, the dopaminergic synapse (Bc = 0.13604918, FDR = 3.86E−08), cAMP signaling pathway (Bc = 0.12989786, FDR = 1.19E−06) and serotonergic synapse (Bc = 0.0838125, FDR = 0.000112) were the nodes with the highest centrality involved in both OCD and BD. The most central molecular functions were dopamine binding (Bc = 0.011851, FDR = 3.59E−10), dopamine receptor activity (Bc = 0.0047987, FDR = 9.72E−09), glutamate-gated ion channel (Bc = 0, FDR = 2.06E−06) and G-protein-coupled amine receptor (Bc = 0.00705387, FDR = 0.000142), respectively. Cellular component enrichment analysis indicated that dendrites (Bc = 0.07980464, FDR = 0.000151) and axon parts (Bc = 0.03089599, FDR = 0.000215) are the main components involved in the pathology of OCD and BD. Finally, it was found that cortex (Bc = 0.22937063, FDR = 0.008), striatum (Bc = 0.1299988, FDR = 0.008) and hippocampus (Bc = 0.07421981, FDR = 0.008) are the main brain regions whose impairments contribute to the pathophysiology of OCD and BD. The other pathways, molecular functions and cellular components are shown in Table 2.

**Table 2:** The results of enrichment analysis for pathways, molecular functions, cellular components and brain regions involved in the pathogenesis of both OCD and BD

	Pathway full name	KEGG ID	FDR	Betweenness centrality
1	Dopaminergic synapse	hsa04728	3.86E−08	0.13604918
2	cAMP signaling pathway	hsa04024	1.19E−06	0.12989786
3	Serotonergic synapse	hsa04726	0.000112	0.0838125
4	Calcium signaling pathway	hsa04020	0.000979	0.07812904
5	Glutamatergic synapse	hsa04724	0.001158	0.04450784
6	GABAergic synapse	hsa04727	0.005543	0.03118593
7	Retrograde endocannabinoid signaling	hsa04723	0.00753	0.04801305

	Molecular functions	Gene ontology (GO) ID	FDR	Betweenness centrality
1	Dopamine binding	GO:0035240	3.59E−10	0.011851
2	Dopamine neurotransmitter receptor activity, coupled via Gs	GO:0001588	9.72E−09	0.0047987
3	Extracellular-glutamate-gated ion channel activity	GO:0005234	2.06E−06	0
4	G-protein-coupled amine receptor activity	GO:0008227	0.000142	0.00705387
5	Amino acid binding	GO:0016597	0.000211	0.02687112
6	Kainate-selective glutamate receptor activity	GO:0015277	0.001517	0
7	Monoamine transmembrane transporter activity	GO:0008504	0.004829	0.00237254
8	Serotonin binding	GO:0051378	0.004829	5.53E−04
9	Ammonium transmembrane transporter activity	GO:0008519	0.028746	0.00237254
10	Serotonin receptor activity	GO:0099589	0.028746	5.53E−04

	Cellular components	Gene ontology (GO) ID	FDR	Betweenness centrality
1	Dendrite	GO:0030425	0.000151	0.07980464
2	Axon part	GO:0033267	0.000215	0.03089599
3	Postsynaptic density	GO:0014069	0.000243	0.05051219
4	Ionotropic glutamate receptor complex	GO:0008328	0.001148	0.0055914
5	Membrane raft	GO:0045121	0.001158	0.05504978
6	Cation channel complex	GO:0034703	0.003335	0.02751275
7	Perikaryon	GO:0043204	0.01023	0.00847745
8	Synaptic vesicle	GO:0008021	0.013692	0.01103456

	Brain regions		FDR	Betweenness centrality
1	Cortex		0.008	0.22937063
2	Striatum		0.008	0.1299988
3	Hippocampus		0.008	0.07421981

## Discussion

The results of enrichment analysis in the present study showed that the genes involved in glutamatergic transmission (*GRIK2*, *GRIK3*, *GRIN2B*, and *GRIA1*), dopaminergic transmission (*DRD1*, *DRD2*, *DRD3*, *DRD4*, and *DRD5*), serotonergic transmission (*SLC6A4*, *HTR1A*, *HTR2A*, and *TPH2*) and GABAergic transmission *(GABBR2*, *GABA*, and *GAD1*) are the most important genes associated with both the disorders.

The calcium voltage-gated channel subunit alpha1 C; *CACNA1C* was found as the most central shared gene. The L-type voltage-gated calcium channel family include four different isoforms consisting of Cav1.1, Cav1.2, Cav1.3, and Cav1.4. CACNA1C codes for the α1C subunit of the Cav1.2 channel is involved in the proper functioning of the hippocampus, amygdala, and mesolimbic reward system circuits, which are strongly implicated in the pathophysiology of psychiatric disorders [40]. While the most statistically robust CACNA1C associations are in BD, polymorphisms in CACNA1C shown to be correlated with other conditions such as schizophrenia, major depressive disorder (MDD), anxiety [41], neuroticism and obsessive–compulsive thoughts [40].

The function of *CACNA1C* gene predicts amygdala and hippocampal activity during emotional processing and hippocampal activation during episodic and working memory recall [42, 43], actions which are suggested as the main diagnostic intermediate phenotypes for both BD and OCD [17]. In humans, *CACNA1C* risk variant modulates an individual’s inclination to respond to reward. The mesolimbic–dopamine system, through the ventral tegmental area (VTA)–nucleus accumbens (NAc) pathway plays a critical role in reward processing and possibly compulsive responses [44]. Stein and Lochner have illustrated that the role of structures related to learning and reward include dopaminergic agonists in OCD [45]. Terrillion et al. have also reported that decreased expression of the *CACNA1C* gene in the mesolimbic pathway reduces mania symptoms [46].

Besides, GRIA1 encodes glutamate receptor, ionotropic, AMPA1, which acts as an excitatory glutamate receptor in the central nervous system. Evidence has suggested that AMPA1 gene is associated with impaired working memory and reward processing in patients with OCD [47]. OCD has been conceptualized as a behavioral addiction with defective processing in reward circuity [48]. Compulsions act as a reward, suppressing the anxiety-provoking obsessions [49]. Also, studies have reported that GRIA1 may regulate the circadian rhythms, through the regulation of *Clock* gene, which has been demonstrated to be disrupted in the ventral tegmental area of patients with BD, particularly in manic episodes [50–52].

DRDR2 is one of the other prominent genes in this collection. The interface between obsessive–compulsive and bipolar disorders can be explained through the concept of “reward deficiency syndrome (RDS)” [53]. The reward deficiency hypothesis proposes that aberrant functioning of normal reward pathways in one individual causes less satisfaction with natural rewards and enhance the inclination to compulsory use of substances or repetitive behaviors as a way to augment stimulation of the reward pathways [54]. Blum et al. assume that addictive, impulsive and compulsive disorders may have a common genetic basis. They point to the role of genes involved in the dopaminergic system in this processes, among which DRD2 is the most determinant gene [55, 56]. A number of independent meta-analyses endorsed the association of DRD2 polymorphisms with RDS [57, 58]. Impulsivity, has been known as a key intermediate phenotype in BD, which is present in inter-episode phases of the illness [59, 60]. This feature has also been recently taken into consideration in OCD [61, 62]. On the other hand, considering the frequent evidences about the risk of impulsivity in suicidal behaviors [63, 64], the presence of impulsivity might consider as a key predictor of suicidal attempts in both OCD and BD. Moreover, DRD2 is associated with learning the motor sequences [65] and the motor components of several psychiatric disorders such as motor defects related to the first episode of psychosis [66].

In the present analysis, dopaminergic synapse as the most significant pathway and dopamine binding and dopamine neurotransmitter receptor activity, as two of the most significant molecular functions have been discovered. These findings point out to the major role of the dopaminergic system in both OCD and BD. Due to the wide-ranging and diverse functions assumed for dopamine, part of these functions supposed to be responsible for dysregulation in structures underlying symptoms of both OCD and BD. These symptoms are contradictory at the level of semiotics and phenotypes. The dopaminergic system and the neurotransmitter dopamine (DA) are responsible for many basic functions such as motivational and emotional behaviors, control of involuntary movements and neurosecretion associated with the biological clock and homeostatic sleep–wake regulation in humans [67]. The DA system also has been known to be impaired in mechanisms involved in motor inhibition [68] and cognitive functions in OCD [69] and BD [70]. Midbrain dopaminergic neurons in the mesocorticolimbic system regulate working memory, attention, decision-making and reward-associated behaviors. Bodea et al. reported that dopamine imbalances in the mesocorticolimbic pathway have been implicated in drug abuse, depression, attention deficit hyperactivity and schizophrenia disorders [71].

Suhara et al. reported that the dopamine-binding potentials in the frontal cortex of the patients with BD were significantly lower than normal controls. Also, Denys et al. demonstrated that the reduced binding potential of the dopamine D receptor, especially the D2 type receptor, is directly involved in the pathophysiology of OCD [72].

Reduced serotonergic activity in the forebrain regions contributes to the development of OCD [73] and in the depression phase of BD [74]. Defective inhibition of GABAergic system in the prefrontal cortex has been also shown in both BD [75] and OCD [76].

Further analysis showed that cAMP signaling pathway is the most central pathway associated with the two disorders. It has been shown that the baseline receptor-mediated level of cAMP in plasma and cerebrospinal fluid is altered in various mood states, including BD and major depression [77]. Perez et al. reported the altered cAMP-dependent kinase activity in platelets of patients with OCD [78].

Moreover, in his well-known hypothesis, Marazziti suggests that OCD is caused by a decreased activity of protein kinase type A (PKA), in the cAMP signaling pathway [79]. In line with Marazziti, Tardito et al. argued that the cAMP-stimulated PKA activity is considerably increased in BD patients compared with healthy controls [80] and cAMP-responsive element-binding (CREB)-1 gene (*CREB1*) is demonstrated to be associated with the risk of BD and obsessive behaviors [81, 82].

On the other hand, transduction of the signal through metabotropic glutamate receptors [83], dopamine receptors [84], and 5-HT1, 4, 5, 6, and 7 receptors [85] are mainly based on the cAMP-mediated cascades. Therefore, the defect in this signal processing system can also affect signal transduction through these receptors, as defects in the glutamatergic, serotonergic, GABAergic and dopaminergic neurotransmission systems in both OCD and BD have been identified [33, 86–88].

Cellular component enrichment analysis revealed that the cellular components involved in receiving the signals, namely, dendrites, were the most important cellular components associated with OCD and BD. In this regard, Rosoklija et al. have indicated the structural abnormalities of dendrites in major mood disorders [89] and Konopaske et al. have found that spine density is significantly reduced in the dorsolateral prefrontal cortex of BD subjects [90].

Finally, our analysis showed that the striatum is the most important brain region associated with obsessive–compulsive and bipolar disorders. Bipolar patients have been reported to show abnormal task-related activity in the striatum [91] and substantial shape alterations in the anterior and ventral striatum [92]. On the other hand, impaired working memory [93], abnormal conditioning [94], and decreased probabilistic learning [95] are all known as striatum-dependent valuable endophenotypes for BD. Differences in volumes of the caudate nucleus and the putamen between OCD patients and healthy controls have been also reported??? [96]. Moreover, functional imaging studies indicated the altered activities in the striatum of OCD patients, both during resting-state and during expression of symptoms [97].

Regarding clinical implications, studies of this kind may help in solving several issues clinicians usually face. If such gene-based infrastructured network is confirmed by future studies, it can be a determining factor in identifying individuals at risk among the siblings of the proband or predicting the risk of developing the disorder in the next generation. On the other hand, prognosis and disease prevention, a focus area for the mental healthcare systems, can be considered. In clinical settings, it is observed that some types of OCD patients tend to develop psychotic or manic symptoms. According to the results, those patients with defects in the most important identified components may be more susceptible to develop symptoms of the other serious illnesses, such as BD. Furthermore, finding more specific and effective drug therapies is the other implication of such findings. For example, in an OCD patient with disturbances in the above network, prescribing mood stabilizers, antipsychotics or other medications which target the identified genes, pathways or molecular functions in addition to SSRIs, may also be recommended prior to the appearance of mania symptoms at the phenotype level.

## Conclusions

Our analysis indicated *CACNA1C*, dopamine receptor binding activity, cAMP signaling pathway, dendrite and striatum as the most central gene, molecular function, pathway, cellular component and brain region, respectively, associated with both OCD and BD. Significantly all these elements are interconnected; in the striatal region, calcium receptor in dendrites may be affected by cAMP-mediated signaling pathways and affect the dopamine receptors, and this network may be the main impaired infrastructure associated with OCD and BD, although confirmation of this hypothesis requires comprehensive and integrated experimental studies in the future. Moreover, considering the functions of the most important genes, pathways, and molecular functions described in this study, it seems that several major functions including the reward processing, motor and cognitive functions such as memory can be pointed out as the most intermediate phenotypes shared between the two disorders. The results of the present study suggest that OCD–BD comorbidity caused by common genes and may occur with exposure to certain environmental factors. Accordingly, it can be assumed that the set of genes involved in the comorbidity of the disorder is different from the set of genes which are involved in the occurrence of each disorder alone. This finding should be further considered and taken into account during diagnoses and pharmacotherapy of the disorders. For example, in cases of comorbidity, it may be useful to prescribe drugs that are not the first line of treatment for either disorder when they occur alone. These findings might also be helpful in estimating the course of any disorder. Further studies could focus on explaining a more precise model of pathogenesis and the role of each component in developing co-occurrence.

## Supplementary information

**Additional file 1.** The name and references of the genes associated with OCD and BD.

## Data Availability

All data analyzed during this study are included in this manuscript and its additional file. The detailed data like the articles from which the data were extracted are available through their references mentioned in additional file or contacting the corresponding author.

## Abbreviations

OCD: Obsessive–compulsive disorder
BD: Bipolar disorder
GSEA: Gene Set Enrichment Analysis
CSEA: Cell Type-Specific Expression Analysis
GWASs: Genome-wide association studies
DA: Neurotransmitter dopamine
*CREB1*: cAMP responsive element-binding (CREB)-1 gene
